# *Dittrichia viscosa* Selection Strategy Based on Stress Produces Stable Clonal Lines for Phytoremediation Applications

**DOI:** 10.3390/plants12132499

**Published:** 2023-06-29

**Authors:** Chiara Anglana, Piergiorgio Capaci, Fabrizio Barozzi, Danilo Migoni, Makarena Rojas, Egidio Stigliano, Francesco Paolo Fanizzi, Gian Pietro Di Sansebastiano, Paride Papadia

**Affiliations:** 1 Department of Biological and Environmental Sciences and Technologies (Di.S.Te.B.A.), University of Salento, Campus Ecotekne, 73100 Lecce, Italy; chiara.anglana@unisalento.it (C.A.); piergiorgio.capaci@unisalento.it (P.C.); fabrizio.barozzi@unisalento.it (F.B.); danilo.migoni@unisalento.it (D.M.); makarena.rojas@unisalento.it (M.R.); fp.fanizzi@unisalento.it (F.P.F.); 2 Green Greener srl, Rione Pascoli 5, 75025 Policoro (MT), Italy; egidiostigliano1979@gmail.com

**Keywords:** *Dittrichia viscosa*, Nip1.1, arsenic, cadmium, phytoremediation

## Abstract

*Dittrichia viscosa* uptake and translocation of the metalloid As is not fully understood and some data are contradictory, but its adaptability to this pollutant is known and is dependent on its genetic variability. *D. viscosa* is not a hyperaccumulator plant, but it can grow in high-drought conditions while still producing large biomass, even tolerating significant concentrations of As^3+^ and As^5+^. In spite of these remarkable characteristics, adaptive modification of performances is not predictable in wild populations. In previous work, we established experimental clonal populations to perform a functional study on the aquaporin NIP1.1. Here, we propose a strategy to select a clonal population of *D. viscosa* with a defined phenotype related to As tolerance and to reduced NIP1.1 expression levels for phytoremediation applications. From the previous work, we selected four independent clones, two of them belonging to the weak population (W8 and W9) and the other two belonging to the strong population (S1 and S3). The weak and strong populations differ for a different expression ratio root/shoot of DvNip1;1 that brings a different tolerance to As presence. The stress response of the populations, revealed by the CAT enzymatic test, was statistically correlated to the clones, but not to As uptake. Performance of the selected plants on a second unrelated metallic pollutant, Cd, was evaluated, showing that Cd uptake is also independent from the tolerant phenotype. In vitro culture methods using solid media and temporary immersion bioreactors were compared to propose an optimized combined protocol. The procedure yielded propagation of genetically stable tolerant clonal lines with good uptake of As and Cd. The plants, mass-produced with the developed in vitro protocol, were able to maintain their acquired abilities and are potentially able be later applied in phytoremediation or contaminated areas’ re-naturalization.

## 1. Introduction

Phytoremediation is the application of plant-controlled interactions with groundwater, soil and organic and inorganic molecules in contaminated sites to achieve site-specific remedial goals [1]. In particular, phytoextraction through the use of specific plants (hyperaccumulators), involving the translocation of contaminants from soil and roots to the above-ground organs, extracts the pollutant from the substrate leading to the phytoremediation of the soil [2].

*Dittrichia viscosa* L. (W. Greuther) (Asteraceae), also known as *Inula viscosa* L., is a very common herbaceous and perennial specie in the Mediterranean region. It is adapted to a wide range of environmental stresses, such as arid and high-salinity land, or invasive anthropic activities (mining or industrial sites). It is an important species with a high ability to grow on polluted soils with metalloids and heavy metals, and it was proposed as a bioindicator plant for these elements. *D. viscosa* has a high potential as a bioaccumulator and it is classified as a good candidate for phytoremediation and phytoextraction [3].

*D. viscosa* spontaneously colonizes contaminated soils and has already been recognized as suitable for metal phytoremediation. Although it is not a hyperaccumulator plant, it can compensate with an exceptional tolerance to adverse growth conditions, (e.g.,: drought) and a biomass larger than hyperaccumulator plants [4].

The metalloid Arsenic (As) and the heavy metal cadmium (Cd) are both very toxic chemical elements and, similarly to other contaminants, are associated with industrial-related lands and to agricultural fields as a consequence of an excessive use of fertilizers, fungicides, herbicides, etc. [5,6,7,8,9,10]. *D. viscosa* is capable of tolerating and remediating soils contaminated with both these pollutants.

While the ability to accumulate and translocate Cd was studied in different experimental conditions [11,12,13], uptake and translocation of As are still not fully understood. 

Pérez-Sirvent et al. [14] reported that *D. viscosa* transfers As from the soil to the root but does not translocate them in large quantities to the aerial parts. Contrarily, Guarino et al. [15] tested different wild clones selected for their capacity to grow on soil polluted with NaAsO_2._ The authors reported As to be fully translocated to *D. viscosa* shoots and then volatilized. In fact, the amount of As present in leaves corresponded only to ca. 0.10–1.7% of the amount supplied, which was no longer detected in the soil. In any case, the data evidenced large variability, among plants, in both As accumulation and translocation.

In a previous work [4], we studied the capacity of *D. viscosa* to uptake, translocate, and grow in different concentrations of As^3+^, As^5+^, and Cd^2+^. In fact, we observed opposite trends in phytoextraction to the aerial part and bioconcentration in the roots for As and Cd. The bioaccumulation capacity for As was inversely correlated with As concentration in the soil. Conversely, the average bioaccumulation for Cd^2+^ was directly proportional to the concentration in the soil. These plants retained As mainly in the roots and translocated it in limited amounts to the aerial parts. Different clonal subpopulations exhibited different behavior, and we selected *D. viscosa* individuals with a better tolerance to As to propagate them in clonal populations for applicative studies and phytoremediation activities.

After that, with the purpose of stabilizing isolated metal-tolerant clones, we performed a functional study on the aquaporin NIP1.1 gene as a marker for arsenic tolerance [16]. The DvNip1 gene was cloned and its expression profile in roots and shoots was characterized under different arsenic stress conditions. The use of clonal lines highlighted that the DvNip1.1 expression level was stably influenced by arsenic stress. The proportion of gene expression in roots and shoots could be used to generate an index that appears to be a promising selection marker to predict arsenic-resistant lines of *Dittrichia viscosa*.

These results were promising, but once selected, plants that are going to be extensively used in the processes of phytoremediation and phytoextraction need rapid multiplication in a short amount of time and with low cost. In vitro micropropagation can be further improved using temporary immersion bioreactors (TIBs). The use of TIBs is a plant-propagation technique, combining the advantages of solid culture media for maximum gas exchanges and liquid culturing for a more efficient nutrient uptake. Temporary immersion improves plant material quality, increasing shoot vigor and the frequency of somatic embryos. Plant growth and proliferation rates are generally stimulated when compared with other propagation techniques. Moreover, it represents a useful tool to study different metabolic processes [17,18]. In vitro propagation of *D. viscosa* with different basal media and phytohormones has been extensively studied in the past [15,19,20], but TIBs have never been used for the propagation of this species. 

In this study, we illustrate a strategy to rapidly propagate a clonal population of *D. viscosa*, previously selected on the base of a diversified NIP1.1 expression level. Different lines were characterized for their tolerance to As and for their ability to uptake As and Cd. More parameters could be explored and characterized accordingly to different desirable applications. Availability of well-characterized clonal plants allows better planning for specific phytoremediation or contaminated areas’ re-naturalization. 

## 2. Results

### 2.1. Dittrichia Viscosa Clonal Populations Maintain Tolerance-Related Phenotype

Plants showing differentiated tolerance to arsenic were selected in a previous study and used to generate a clonal population. Some showed a weak (W) phenotype, while others showed a strong (S) phenotype when treated with As^3+^ [4]. Specifically, in this study, W8, W9, S1, and S3 plant lines were chosen [16] (Supplementary Figure S1). These clonal populations were maintained in vitro for 2 years with periodical tips subculturing on hormone-free MS medium.

**Figure 1:**
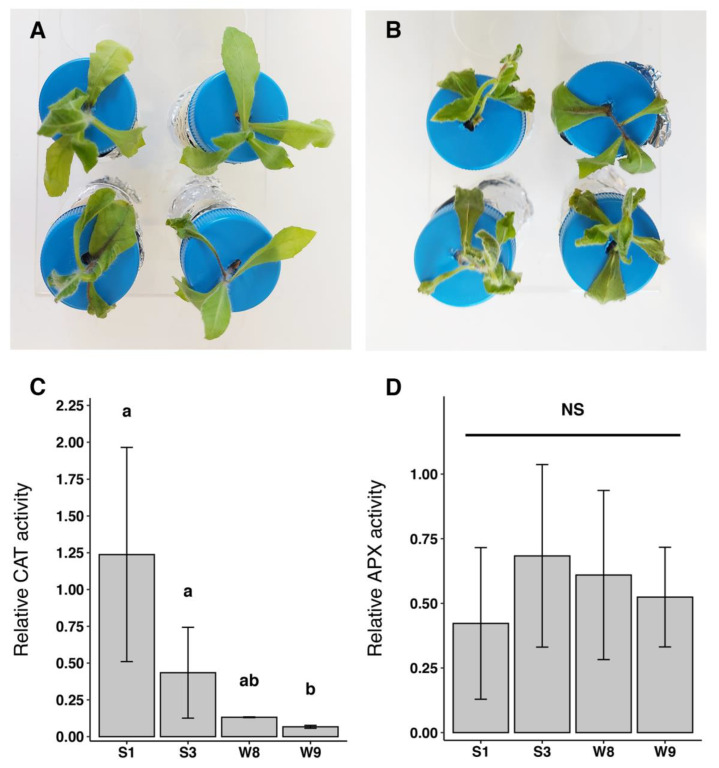
Hydroponically grown *D. viscosa* plants differently tolerate As^3+^ 90 µM and Cd^2+^ 100 µM 3-day treatment. (**A**) S plants; (**B**) W plants. (**C**) Increase in CAT activity relative to control; (**D**) increase in APX activity relative to control. Statistical analysis by Kruskal–Wallis test with Bonferroni post-hoc test (*p* = 0.05). Bars with same letters are not significantly different. NS = non statistically significant difference. Vertical bars show standard deviation. n ≥ 3.

Clones of the lines selected to have a strong phenotype for As generally maintained turgor for the time of the experiment (Figure 1A); clones of the lines selected as having a weak phenotype suffered evident higher stress and turgor loss (Figure 1B). Abiotic stress in the treated plants was measured with two independent tests, CAT (catalase activity) and APX (ascorbate peroxidase activity), showing partial correlation with phenotype. CAT activity expressed as an index of CAT activity in stressed plants over the activity in unstressed controls, appeared significantly different in S plants compared to W plants (Figure 1C). Strong plants showed stronger CAT activity relative to controls. APX activity, on the contrary, appeared similarly reduced in all clonal populations (Figure 1D).

### 2.2. D. viscosa Clonal Individuals Accumulate HMs and Metalloids Independently from Phenotype

**Figure 2:**
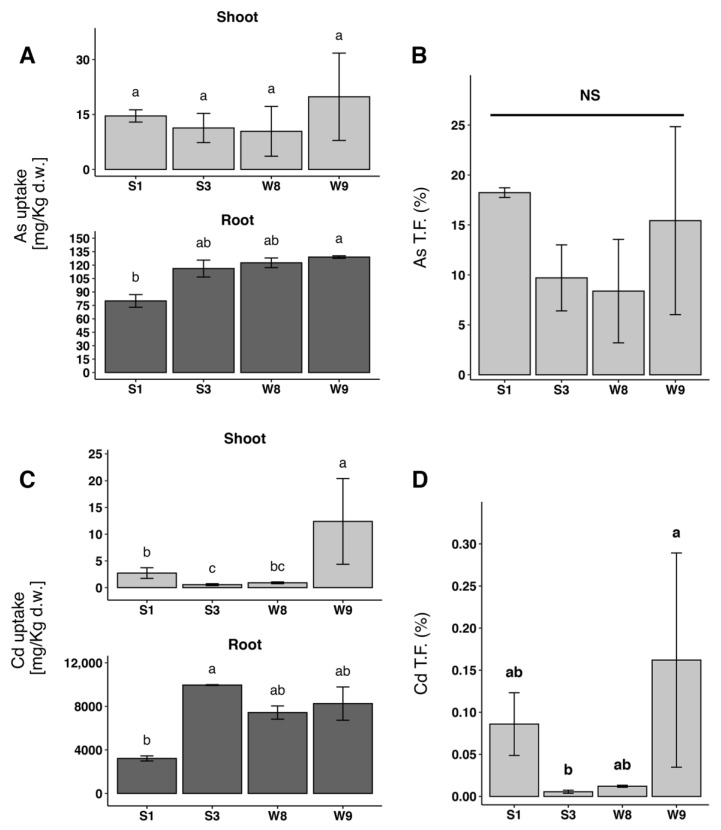
Accumulation in shoots and roots of As and Cd with relative T.F. (**A**) As accumulation in shoots and roots; (**B**) As T.F.; (**C**) Cd accumulation in shoots and roots; (**D**) Cd T.F. statistical analysis by Kruskal–Wallis test with Bonferroni post-hoc test (*p* = 0.05). Bars with same letters are not significantly different. NS = non statistically significant difference. Vertical bars show standard deviation. n ≥ 3.

After treatment, the plants were separated in shoots and roots and washed accurately, dried and analyzed using inductively coupled plasma atomic emission spectroscopy (ICP/AES). Regarding As uptake, no differences with statistical relevance in the organs of W or S plants related to the different phenotype were observed (Figure 2A). The translocation factor (T.F.) was not significantly different either (Figure 2B). It was not possible to relate Cd accumulation in the different organs to the W or S phenotype (Figure 2C). Additionally, the T.F. did not show any correlation to phenotypic groups (Figure 2D).

### 2.3. Optimization of D. viscosa Micropropagation: Effect of TIB and Solid Culture Medium on Length, Multiplication Rate, Fresh Weight, Rooting of Explants

*D. viscosa* is easily propagated vegetatively, even under nonsterile conditions, but for effective use in phytoremediation of adapted plants, process optimization in space and time is important. Two completely different approaches were used to micropropagate a single, previously established [16] clonal plant population in vitro. A system based on TIBs and a more conventional approach using solid culture medium (SCM) was employed.

**Figure 3:**
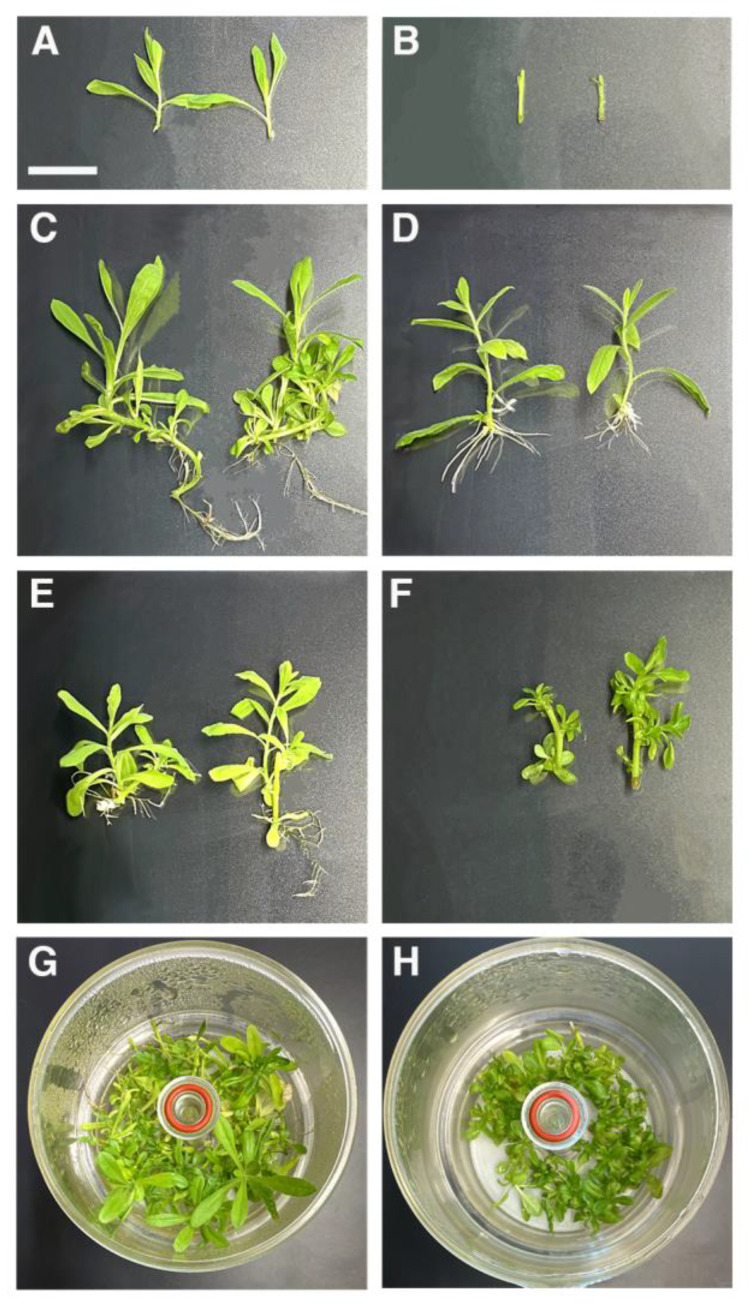
In vitro proliferation of *D. viscosa* explants in SCM and RITA using hormone-free ½ MS medium, 0.5 g L^−1^ sucrose and 0.1% PPM after 45 days of cultivation (scale bar = 20 mm). (**A**,**B**) Initial explants: shoots and nodal segments, respectively; (**C**) shoot cultivated and rooted in RITA with branching; (**D**) shoot growth and shoot rooted on SCM; (**E**) shoot developed and elongated from node cultivated in SCM; (**F**) shoots scarcely developed from nodal segments incubated in RITA; (**G**,**H**) General view of shoots and node cultivated in RITA after 45 days.

**Figure 4:**
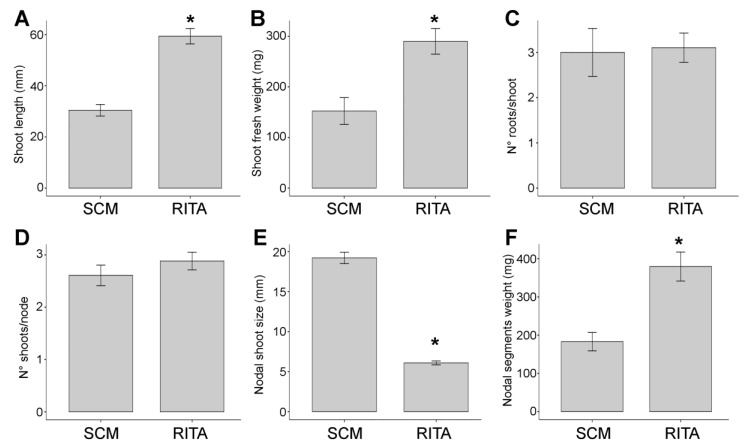
Effect of the TIB and solid culture medium on shoot tips: length (**A**), fresh weight (**B**) and number of roots per shoot (**C**); on nodal segments: number of shoots per node (**D**), shoots size (**E**) and fresh weight (**F**). Statistical analysis by one-way ANOVA with Tukey post-hoc test (*p* < 0.001 indicated by *). The data represent mean values ± standard error of the mean. n > 25.

We found that the addition of phytohormones did not directly benefit the subculture of *D. viscosa* nodal segments. In fact, the addition of kinetins inhibited branching, and the addition of 2-iP and BAP resulted in the induction of abnormal shoots, as previously described by Boonne et al. [19]. After confirming these findings, we decided to use a simple and economical medium with half MS macro- and microelements, sucrose reduced to 0.5% and no growth regulators. Both shoot tips and nodal segments were used without a significant loss of vitality. The two in vitro culture systems had a different effect on length and fresh weight of explants (Figure 3 and Figure 4).

The initial explants (shoots and node, respectively, Figure 3A,B) showed different behavior in growth and development during 45 days of incubation in RITA and SCM. Specifically, cultivation of shoots in RITA resulted in greater development vigor and branching (Figure 3C) than those in SCM (Figure 3D), which exhibited lower growth.

SCM promoted nodal shoot size and each node produced at least two shoots (>17 mm,) in 45 days (Figure 3E). The TIB promoted low shoot elongation from the node, and shoot length was less than those grown in SCM (Figure 4F). In addition, any morphological disorders, such as hyperhydricity or leaves wrinkling, were observed in the shoots and nodes culture in the RITA system after 45 days (Figure 3G,H).

In terms of shoot tip length, the TIB promoted a significant increase in length with a difference of almost 30 mm compared to SCM (Figure 4A). Changes in fresh weight confirmed better performance of TIBs (Figure 4B). In addition, unlike SCM, the TIB induced root sprouting and active root growth after only 7 days of incubation, whereas SCM did not produce the first roots for 12 days. However, no significant difference was observed in the number of roots formed by shoots in the TIB and SCM (Figure 4C).

The number of shoots per node did not vary significantly in both culture systems (Figure 4D). On the other hand, SCM proved to be more suitable for shoot development from nodal segments than the TIB (Figure 4E). Although the length of the shoots regenerated from nodes was higher in SCM, nodal segments in the TIB showed a significant difference in fresh weight (Figure 4F).

### 2.4. Establishment of an Efficient Micropropagation Process Using SCM and TIB 

**Figure 5:**
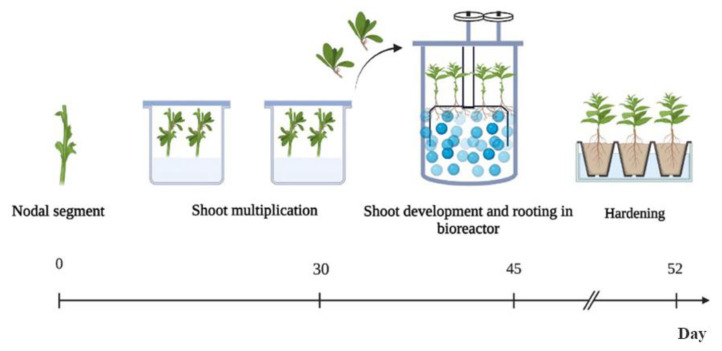
Micropropagation protocol for *Dittrichia viscosa* based on initial shoot multiplication on SCM, followed by shoot development and rooting of shoots in a bioreactor.

The experimental approach evidenced that culture in the TIB boosted the length of isolated shoots, their fresh weight and root formation but was not sufficient to stimulate shoot elongation from nodes, which are the most common source of preformed buds in plants. To scale-up micropropagation of *D. viscosa*, SCM and TIB were then combined in three steps (Figure 5).

Nodal explants were first incubated in solid medium for shoot propagation and initial growth. The growing shoots, approximately 13 mm in size, were cut after 30 days (before the optimal time for transfer to SCM) and transferred to TIB, which guarantees better elongation and rooting. This step was also useful to improving plant acclimation. Finally, after only 15 days, elongated and rooted shoots were transplanted into rock wool ex vitro and placed into Steri Vent high model containers for the first 5 days to prevent rapid dehydration. 

## 3. Discussion

Plant traits’ transgenerational inheritance for adaptation to environmental changes is known. In a study on different pine trees species, the hypothesis that abiotic stress in the maternal environment can induce an inherited “transgenerational plasticity” was proven [21]. The potential of this capacity for practical applications is easy to imagine [22]. We started from the concept that the selection of optimized plants for phytoremediation can be customized in a laboratory and prepared for field use.

Here, we showed that we were able to isolate, propagate, and molecularly characterize a large clonal population of *Dittrichia* plants optimized for arsenic phytoremediation, starting from a collection of previously produced and partially characterized clones [4,16]. We selected two clones with a tolerant (strong = S) phenotype and two clones with a sensitive (weak = W) phenotype that maintained their phenotype in the absence of As treatments for over two years of in vitro propagation.

Abiotic stress in the treated plants was measured with two enzymatic activity tests, CAT (catalase activity) and APX (ascorbate peroxidase activity).

When plants are exposed to stress, reactive oxygen species (ROS) production increases and can damage the cell. A decrease in CAT activity occurs in various plant species due to oxidative stress and seems to be related to salicylic acid accumulation [23]. The importance of CAT in scavenging active oxygen generated under stress conditions was related to several oxidative stresses, such as salt, ozone, herbicides and H_2_O_2_ treatments. However, a decrease in CAT activity could be observed under some stress conditions that, on the contrary, continue to induce other enzymes of the active oxygen species-scavenging system, such as SOD, APX and GR [24].

That being said, the major hydrogen peroxide detoxifying system is the ascorbate-glutathione cycle, in which ascorbate peroxidase (APX) enzymes play a key role. It catalyzes the conversion of H_2_O_2_ into H_2_O, using ascorbate as a specific electron donor. The importance of APX is related but not restricted to chloroplasts. It also scavenges ROS in the cytosol, mitochondria and peroxisomes [25].

The different ROS-scavenging enzymes in plants, such as CAT, SOD and GSH reductase (GR), increase in parallel [10,26]. In a recent study [27] in which *Dittrichia* is challenged with Thallium(I), the authors showed that APX activity in leaves was just slightly increased by increasing concentrations of the HM, with a resulting H_2_O_2_ concentration increment suggesting a weak increase in CAT activity. Also, GR activity did not change with the increasing concentration of HM. Taking these considerations all together, we can argue either that the antioxidant enzymes analyzed are not involved in the determination of the phenotype, or that the plants have yet to adapt to the stress and do not show differences when compared to the non-stressed condition.

In order to investigate As and Cd accumulation and translocation, we used the ICP/AES method. The analysis showed that no differences in the accumulation or in the T.F. of As and Cd had statistical relevance in the organs of W or S plants (Figure 2). We already showed that tolerance to As is not related to a reduced uptake. In fact, the null mutation of the aquaporin Nip1.1 gene in Arabidopsis makes the plant fully resistant to the metalloid without preventing accumulation [28,29]. This aquaporin, localized in the Endoplasmic Reticulum is responsible for arsenite (As^3+^) [30] and antimony (Sb) [31] membrane permeation, but the uptake of As^3+^ in the null mutant [28,29] suggests a sophisticated mode of action where endomembranes and compartmentalization in the cells are involved, since other elements accumulation can also be altered [28]. The involvement of aquaporins in the adaptive transformation of endomembranes is not a unique finding. Ariani and co-workers [32] showed that excess of Zn down-regulates another NIP, aqua1, affecting the formation of new pro-vacuoles. 

These observations prompt us to accept the evidence that plants can adapt to arsenic contamination through complex mechanisms that can be furthermore inherited by vegetatively propagated individuals, but these mechanisms still require deep characterization.

In our case, we intended to evaluate the possibility of selecting an improved line of plants to phytoremediate As-polluted areas. We were able to conclude that the clone S1 appears to be the best candidate as it has a strong phenotype, is tolerant to As^3+^ and is still able to accumulate and translocate the metalloid with the best T.F.

Moreover, the clonal population herein characterized was propagated for 2 years, proving that adaptation to a selective agent or a toxic pollutant can be maintained for extended periods. Nonetheless, in the case of a study focused on selecting plants with optimized performance to phytoremediate specific pollutants, the goal becomes to vegetatively propagate the selected plant rapidly and in large quantities.

Vegetative propagation of plants can be dramatically accelerated by in vitro culture. The first micropropagation protocol for *D. viscosa* was developed by [19] using nodal explants. We used solid media and temporary immersion bioreactors to optimize *D. viscosa* propagation without any hormonal treatment and limiting the use of nutrients to limit the process cost.

Plants obtained from nodal explants are less prone to genetic variation, thus justifying the increased use of nodes in micropropagation [15]. Since TIB systems usually appear very beneficial to plant regeneration, we first tried to stimulate shoot formation from nodes in RITA units. The efficacy of these systems needs to be optimized depending on the plant species propagated and, sometimes, on the kind of explant used within the same species [33]. For instance, Mordocco et al. [34] reported that sugarcane leaf explants failed to induce shoots when placed directly in RITA units. We also observed that placing nodes in RITA failed to stimulate shoot development. Shoot multiplication in SCM was required in *Dittrichia viscosa* to achieve high shoot development and rooting in TIB. The environment provided by a solid medium, such as a slow and continuous supply of plant growth regulators and nutrients, adequate osmotic conditions and tissue polarity, likely guarantees meristem formation and growth during the initial culturing phases, which is considered the most critical [34]. Despite the fact that the labor component could be reduced and the intensification of culture could be increased with automated TIBs [18], establishing a free contaminated culture is a significant obstacle in the initial phase, and the use of SCM is preferable. Our results confirmed that TIBs can be used for propagation, but their use has to be inserted in a more complex procedure in which TIBs’ main role is the stimulation of root formation.

The two in vitro culture system combined semi-automation of the micropropagation: incubation of nodal segments in SCM, followed by the rapid development and rooting of shoots in an RITA bioreactor (Figure 4) [18].

Using combined SCM and TIBs, a three-fold multiplication of plantlets could be obtained in 52 days, saving on time, labor and costs. In fact, starting with only 5 plants in vitro, it was possible to excise 10 nodal segments without sacrificing the mother plant (which could be partially cut off and reused to produce additional shoots). About 25 plantlets could be grown from these explants. The procedure multiplied the number of plants by three-fold in 52 days, without the need to use growth regulators.

On the other hand, when starting from the shoot tips in TIBs, there was no real increase in the number of plants despite the very short rooting time (7–10 days). The excised plant could regenerate one or two shoot tips in a variable period of 1 to 3 weeks to allow a new round of shoot tips’ excision. This was probably due to the plagiotropic position of shoot tips in the bioreactor and could provide an additional source of explants. In any case, elongation is the most efficient growth mode for shoot tips in TIBs, and the system can be better used to produce nodal segments providing multiple rounds of propagation in a shorter time.

In conclusion, in this work, we demonstrated how it is possible to select the plant through specific treatments that influence plant epigenetic regulation and propagate it, maintaining the desired characteristics. We used As stress tolerance as a selection marker because of the great importance of this kind of contamination, demanding new phytoremediation tools [35]. We also characterized a relevant independent parameter, such as Cd uptake, since it is important to also consider the independent segregation of characters different from the one object of selection. Finally, we proposed a protocol involving TIB technology to perform in vitro mass production of the improved plants since their application in phytoremediation or contaminated areas’ re-naturalization require the establishment of a complete applicative strategy.

## 4. Materials and Methods

### 4.1. Plant Material for Chemicals Uptake Assay 

For metal uptake analysis, plants with weak (W) and strong (S) tolerance to As^3+^ were selected from a previous study [4]. Specifically, tips from W8, W9, S1 and S3 plants [16] propagated in vitro for 2 years were cut and hydroponically grown to induce the formation of adventitious roots. The plant growth medium used was based on one-tenth-strength modified Hoagland’s solution [0.28 mM Ca(NO_3_)_2_, 0.5 mM KNO_3_, 0.2 mM MgSO_4_, 0.1 mM NH_4_NO_3_, 0.1 mM KH_2_PO_4_, 5 µM Fe-EDTA, 4.63 µM H_3_BO_3_, 32 nM CuSO_4_, 915 nM MnCl_2_, 77nM ZnSO_4_, 11 nM (NH_4_)_6_Mo_7_O_24_ · 4H_2_O]. After 14 days, the rooted tips were treated in fresh Hoagland media for 24 h with a combination of As^3+^ (NaAsO_2_) and Cd^2+^ (Cd(NO_3_)_2_) at concentrations of 90 µM and 100 µM, respectively. After treatment, the plants were separated into shoots and roots and washed one time in double-distilled water, two times in 20 mM CaCl_2_, one time in 10 mM EDTA (pH 5.7) and one time again in double-distilled water. Each washing step was carried out using cold solution at 4 °C for 10 min using a tube rotator disk. After washing, the samples were dried at 50 °C.

### 4.2. CAT and APX Activity Measurement

Plants were treated for a 3-day-long period, waiting for the appearance of phenotypic differences. A total of 0.2 g of shoot apex (leaves and steam) hydroponically growth was ground using a chilled potter directly in 1.0 mL extraction buffer (50 mM potassium phosphate buffer (pH 7.6), 0.1 mM EDTA, 0.1% Triton X-100 and 0.5 mM PMSF). After homogenization, the extract was centrifuged at 15,000× *g* for 20 min. The supernatant was collected in a fresh tube and used as the crude enzyme solution.

Catalase activity (CAT) was assayed according to the procedure of Aebi [36], by monitoring the rate of decomposition of H_2_O_2_ at 240 nm in the reaction mixture (3.0 mL), consisting of 50 mM potassium phosphate buffer (pH 7.0), 10 mM H_2_O_2_ and 10 µL of crude enzyme solution.

Ascorbate peroxidase (APX) activity was assayed according to Elavarthi and Martin [37] by monitoring the rate of decomposition of ascorbate at 290 nm. The reaction mixture (2.0 mL) contained 50 mM potassium phosphate buffer (pH 7.0), 0.5 mM H_2_O_2_, 0.5 mM ascorbate and 20 µL crude enzyme solution.

For both activity measurements, the decrease in absorbance was monitored for 3 min and expressed in nmol (H_2_O_2_ or ascorbate)/min/mg of proteins in the crude enzyme solution. The amount of proteins in the crude enzyme solution was determined using Lowry assays [38].

### 4.3. Samples Mineralization and ICP Analysis

The dry weight of the roots and shoots was determined, and the samples were mineralized as previously described [28]; samples were moved to a PP tube and 1 mL of HNO_3_ (ICP grade) was added. The tubes were moved to a water bath at 85 °C. After 15 min, 1 mL of H_2_O_2_ (ICP grade) was added and the samples were incubated at 85 °C for 2 h. At the end of mineralization, the volume was adjusted to 10 mL with ICP grade water and analyzed using ICP/AES.

### 4.4. Micropropagation in Solid and TIB Culture Systems

Shoot tips (about 17 mm long) and nodal segments (about 13 mm) from DI3-F in vitro grown plants [4,16] were used as the source of explants. For the proliferation of shoots, rooting and biomass production, two in vitro culture systems were used: solid and liquid (RITA^®^ system, VITROPIC, Saint-Mathieu-de-Tréviers, France). For solid media, 23 explants (shoot tips and nodal segments) were individually placed in culture tubes “De Wit” (height 130 mm; diameter middle 27 mm; and diameter bottom 10 mm) containing 10 mL hormone-free MS (Murashige and Skoog, 1962) medium with the nutrients reduced to half (1/2 MS) and supplemented with 0.5 g/L sucrose, 8 g/L agar (Duchefa Biochemie) and 0.1% PPM (Plant Preservative Mixture, produced by Plant Cell Technology, Inc. Washington, DC, USA). pH was adjusted to 5.7 before autoclaving (121 °C, 1 atm) for 16 min. 

Cultures were incubated at 25 ± 2 °C under a 16 h photoperiod and transferred to a fresh medium every 14 days.

The rooted explants were washed with tap water and planted in rock wool, which was placed into Steri Vent high model containers (height 96 mm). The plants were fertilized weekly with 75 mL of Hoagland solution adjusted to pH 5.7. The survival rates, length (mm), fresh weight and dry weight after three weeks were recorded. 

For TIB (temporary immersion bioreactor) culture systems, shoot tips and nodal segments were excised from plants obtained in solid medium cultures. Cultures were grown in two RITA^®^ units with 200 mL of liquid MS medium with the same composition as the solid medium. Each RITA^®^ unit contained 15 explants of uniform size and all cultures were kept at 25 ± 2 °C under a 16 h photoperiod. Explants were immersed for 3 min every 8 h. 

The solid medium and liquid/TIB cultures were conducted in parallel from the same kind of starting material. The two experimental approaches were organized over a six weeks’ period. 

Data refer to length (mm) and fresh weight (mg) of shoots, or number of roots per shoots. Concerning nodal segments, the number of shoots per explants, nodal shoot size (mm) and fresh weight (mg) were recorded. 

### 4.5. Statistical Analysis 

Data were analyzed using R software (R Core Team, 2023) [39], R studio (RStudio Team, 2020) [40] and ImageJ [41].

## Data Availability

Data are available on request from the authors.

## Supplementary Materials

The following supporting information can be downloaded at: https://www.mdpi.com/2223-7747/12/13/2499/s1, Figure S1 Relative expression level of DvNip1;1 in the used clones.

## References

[1] Landmeyer J.E. (2012). Introduction to Phytoremediation of Contaminated Groundwater.

[2] Pandey J., Verma R.K., Singh S. (2019). Suitability of Aromatic Plants for Phytoremediation of Heavy Metal Contaminated Areas: A Review. Int. J. Phytoremediation.

[3] Parolin P., Scotta M.I., Bresch C. (2014). Biology of *Dittrichia viscosa*, a Mediterranean Ruderal Plant: A Review. Phyton-Int. J. Exp. Bot..

[4] Papadia P., Barozzi F., Angilé F., Migoni D., Piro G., Fanizzi F.P., Di Sansebastiano G.-P. (2020). Evaluation of *Dittrichia Viscosa* Performance in Substrates with Moderately Low Levels of As and Cd Contamination. Plant Biosyst. Int. J. Deal. All Asp. Plant Biol..

[5] Hu W., Wang H., Dong L., Huang B., Borggaard O.K., Bruun Hansen H.C., He Y., Holm P.E. (2018). Source Identification of Heavy Metals in Peri-Urban Agricultural Soils of Southeast China: An Integrated Approach. Environ. Pollut..

[6] Lv J. (2019). Multivariate Receptor Models and Robust Geostatistics to Estimate Source Apportionment of Heavy Metals in Soils. Environ. Pollut..

[7] Shi T., Ma J., Wu F., Ju T., Gong Y., Zhang Y., Wu X., Hou H., Zhao L., Shi H. (2019). Mass Balance-Based Inventory of Heavy Metals Inputs to and Outputs from Agricultural Soils in Zhejiang Province, China. Sci. Total Environ..

[8] Migoni D., Papadia P., Cannito F., Fanizzi F.P. (2021). Sequential Extraction Analysis of Arsenic in Soil Samples Collected in an Agricultural Area of Brindisi, Apulia (Italy), in the Proximity of a Coal-Burning Power Plant. Appl. Sci..

[9] De Benedictis M., Gallo A., Migoni D., Papadia P., Roversi P., Santino A. (2023). Cadmium Treatment Induces Endoplasmic Reticulum Stress and Unfolded Protein Response in Arabidopsis Thaliana. Plant Physiol. Biochem..

[10] Ghuge S.A., Nikalje G.C., Kadam U.S., Suprasanna P., Hong J.C. (2023). Comprehensive Mechanisms of Heavy Metal Toxicity in Plants, Detoxification, and Remediation. J. Hazard. Mater..

[11] Barbafieri M., Dadea C., Tassi E., Bretzel F., Fanfani L. (2011). Uptake of Heavy Metals by Native Species Growing in a Mining Area in Sardinia, Italy: Discovering Native Flora for Phytoremediation. Int. J. Phytoremediation.

[12] Jiménez M.N., Bacchetta G., Casti M., Navarro F.B., Lallena A.M., Fernández-Ondoño E. (2011). Potential Use in Phytoremediation of Three Plant Species Growing on Contaminated Mine-Tailing Soils in Sardinia. Ecol. Eng..

[13] Buscaroli A., Zannoni D., Menichetti M., Dinelli E. (2017). Assessment of Metal Accumulation Capacity of *Dittrichia viscosa* (L.) Greuter in Two Different Italian Mine Areas for Contaminated Soils Remediation. J. Geochem. Explor..

[14] Pérez-Sirvent C., Martínez-Sánchez M.J., Martínez-López S., Bech J., Bolan N. (2012). Distribution and Bioaccumulation of Arsenic and Antimony in *Dittrichia Viscosa* Growing in Mining-Affected Semiarid Soils in Southeast Spain. J. Geochem. Explor..

[15] Guarino F., Conte B., Improta G., Sciarrillo R., Castiglione S., Cicatelli A., Guarino C. (2018). Genetic Characterization, Micropropagation, and Potential Use for Arsenic Phytoremediation of *Dittrichia viscosa* (L.) Greuter. Ecotoxicol. Environ. Saf..

[16] De Paolis A., De Caroli M., Rojas M., Curci L.M., Piro G., Di Sansebastiano G.-P. (2022). Evaluation of *Dittrichia viscosa* Aquaporin Nip1.1 Gene as Marker for Arsenic-Tolerant Plant Selection. Plants.

[17] Etienne H., Berthouly M. (2002). Temporary Immersion Systems in Plant Micropropagation. Plant Cell Tissue Organ Cult..

[18] Georgiev V., Schumann A., Pavlov A., Bley T. (2014). Temporary Immersion Systems in Plant Biotechnology. Eng. Life Sci..

[19] Boonne C., Wacquant J.P., Jonard R. (1992). In-Vitro Cloning of *Dittrichia viscosa* for Screening Nutritional Ecotypes. Plant Soil.

[20] Romano A. (1999). Callus Induction and Micropropagation of *Dittrichia viscosa* (L.) W. Greuter. II WOCMAP Congress Medicinal and Aromatic Plants, Part 3: Agricultural Production, Post Harvest Techniques, Biotechnology.

[21] Arencibia A., Rodriguez C., Roco L., Vergara C., Gonzalez-Soto N., Garcia-Gonzalez R. (2016). Tolerance to Heavy Metal Stress in Seedlings of Three Pine Species from Contrasting Environmental Conditions in Chile. iForest Biogeosci. For..

[22] Hauser M.-T., Aufsatz W., Jonak C., Luschnig C. (2011). Transgenerational Epigenetic Inheritance in Plants. Biochim. et Biophys. Acta Gene Regul. Mech..

[23] Shim I.-S., Momose Y., Yamamoto A., Kim D.-W., Usui K. (2003). Inhibition of Catalase Activity by Oxidative Stress and Its Relationship to Salicylic Acid Accumulation in Plants. Plant Growth Regul..

[24] Mishra N.P., Mishra R.K., Singhal G.S. (1993). Changes in the Activities of Anti-Oxidant Enzymes during Exposure of Intact Wheat Leaves to Strong Visible Light at Different Temperatures in the Presence of Protein Synthesis Inhibitors. Plant Physiol..

[25] Caverzan A., Passaia G., Rosa S.B., Ribeiro C.W., Lazzarotto F., Margis-Pinheiro M. (2012). Plant Responses to Stresses: Role of Ascorbate Peroxidase in the Antioxidant Protection. Genet. Mol. Biol..

[26] Shigeoka S., Ishikawa T., Tamoi M., Miyagawa Y., Takeda T., Yabuta Y., Yoshimura K. (2002). Regulation and Function of Ascorbate Peroxidase Isoenzymes. J. Exp. Bot..

[27] Espinosa F., Ortega A., Espinosa-Vellarino F.L., Garrido I. (2023). Effect of Thallium(I) on Growth, Nutrient Absorption, Photosynthetic Pigments, and Antioxidant Response of *Dittrichia* Plants. Antioxidants.

[28] Barozzi F., Papadia P., Stefano G., Renna L., Brandizzi F., Migoni D., Fanizzi F.P., Piro G., Di Sansebastiano G.-P. (2019). Variation in Membrane Trafficking Linked to SNARE AtSYP51 Interaction with Aquaporin NIP1;1. Front. Plant Sci..

[29] Ji R., Zhou L., Liu J., Wang Y., Yang L., Zheng Q., Zhang C., Zhang B., Ge H., Yang Y. (2017). Calcium-Dependent Protein Kinase CPK31 Interacts with Arsenic Transporter AtNIP1;1 and Regulates Arsenite Uptake in *Arabidopsis thaliana*. PLoS ONE.

[30] Kamiya T., Tanaka M., Mitani N., Ma J.F., Maeshima M., Fujiwara T. (2009). NIP1;1, an Aquaporin Homolog, Determines the Arsenite Sensitivity of *Arabidopsis thaliana*. J. Biol. Chem..

[31] Kamiya T., Fujiwara T. (2009). Arabidopsis NIP1;1 Transports Antimonite and Determines Antimonite Sensitivity. Plant Cell Physiol..

[32] Ariani A., Barozzi F., Sebastiani L., di Toppi L.S., di Sansebastiano G.P., Andreucci A. (2019). AQUA1 Is a Mercury Sensitive Poplar Aquaporin Regulated at Transcriptional and Post-Translational Levels by Zn Stress. Plant Physiol. Biochem..

[33] Akdemir H., Süzerer V., Onay A., Tilkat E., Ersali Y., Çiftçi Y.O. (2014). Micropropagation of the Pistachio and Its Rootstocks by Temporary Immersion System. Plant Cell Tissue Organ Cult..

[34] Mordocco A.M., Brumbley J.A., Lakshmanan P. (2009). Development of a Temporary Immersion System (RITA^®^) for Mass Production of Sugarcane (*Saccharum* spp. Interspecific Hybrids). Vitr. Cell. Dev. Biol. Plant.

[35] Sinha D., Datta S., Mishra R., Agarwal P., Kumari T., Adeyemi S.B., Kumar Maurya A., Ganguly S., Atique U., Seal S. (2023). Negative Impacts of Arsenic on Plants and Mitigation Strategies. Plants.

[36] Aebi H. (1984). [13] Catalase In Vitro. Methods in Enzymology.

[37] Elavarthi S., Martin B., Sunkar R. (2010). Spectrophotometric Assays for Antioxidant Enzymes in Plants. Plant Stress Tolerance: Methods and Protocols.

[38] Lowry O.H., Rosebrough N.J., Farr A.L., Randall R.J. (1951). Protein Measurement with the Folin Phenol Reagent. J. Biol. Chem..

[39] R Core Team (2023). R: A Language and Environment for Statistical Computing.

[40] RStudio Team (2020). RStudio: Integrated Development Environment for R.

[41] Schneider C.A., Rasband W.S., Eliceiri K.W. (2012). NIH Image to ImageJ: 25 Years of Image Analysis. Nat. Methods.

