# Correction: Anglana et al. *Dittrichia viscosa* Selection Strategy Based on Stress Produces Stable Clonal Lines for Phytoremediation Applications. *Plants* 2023, *12*, 2499

**DOI:** 10.3390/plants12233973

**Published:** 2023-11-25

**Authors:** Chiara Anglana, Piergiorgio Capaci, Fabrizio Barozzi, Danilo Migoni, Makarena Rojas, Egidio Stigliano, Francesco Paolo Fanizzi, Gian Pietro Di Sansebastiano, Paride Papadia

**Affiliations:** 1 Department of Biological and Environmental Sciences and Technologies (Di.S.Te.B.A.), University of Salento, Campus Ecotekne, 73100 Lecce, Italy; chiara.anglana@unisalento.it (C.A.); piergiorgio.capaci@unisalento.it (P.C.); fabrizio.barozzi@unisalento.it (F.B.); danilo.migoni@unisalento.it (D.M.); makarena.rojas@unisalento.it (M.R.); fp.fanizzi@unisalento.it (F.P.F.); 2 Green Greener srl, Rione Pascoli 5, 75025 Policoro (MT), Italy

## Error in Authors’ List

In the original publication [1], there was a mistake in the list of authors. A correction has been made to the list of authors (Page 1):

Chiara Anglana ^1,†^, Piergiorgio Capaci ^1,†^, Fabrizio Barozzi ^1,†^, Danilo Migoni ^1^, Makarena Rojas ^1^, Egidio Stigliano ^2^, Francesco Paolo Fanizzi ^1^, Gian Pietro Di Sansebastiano ^1,^*, Paride Papadia ^1,^*.

## Text Correction

A correction has been made to the Author Contributions (Page 11):

Author Contributions: C.A., P.C., F.B., P.P. and G.P.D.S.: equally contributed to the study design and implementation. Material preparation and data collection were performed by C.A., P.C., F.B., M.R. and F.P.F. provided the reagents and the facilities used for all the chemical analysis. Chemical analysis were performed by F.B. and D.M. The first draft of the manuscript was written by P.P. and G.P.D.S. The manuscript was critically reviewed by F.P.F., C.A., P.C., F.B., and E.S. All authors have read and agreed to the published version of the manuscript.

The authors state that the scientific conclusions are unaffected. This correction was approved by the Academic Editor. The original publication has also been updated.
