# Supplementary material for: Dittrichia viscosa Selection Strategy Based on Stress Produces Stable Clonal Lines for Phytoremediation Applications
Source: Plants (Basel). 2023 Jun 29;12(13):2499. doi: 10.3390/plants12132499 (PMC10346588; doi:10.3390/plants12132499)
Supplement: Supplementary file 1 [file plants-12-02499-s001.zip › plants-2430049-supplementary.pdf]

Article

Dittrichia viscosa Selection Strategy Based on Stress Produces Stable Clonal Lines for Phytoremediation Applications

Chiara Anglana[1,†], Piergiorgio Capaci[1,†], Fabrizio Barozzi[1,†], Danilo Migoni[1], Makarena Rojas[1],Egidio Stigliano[2], Gian Pietro Di Sansebastiano[1,*], Paride Papadia[1,*]

Figure S1: Relative expression level of DvNip1;1 in the used clones

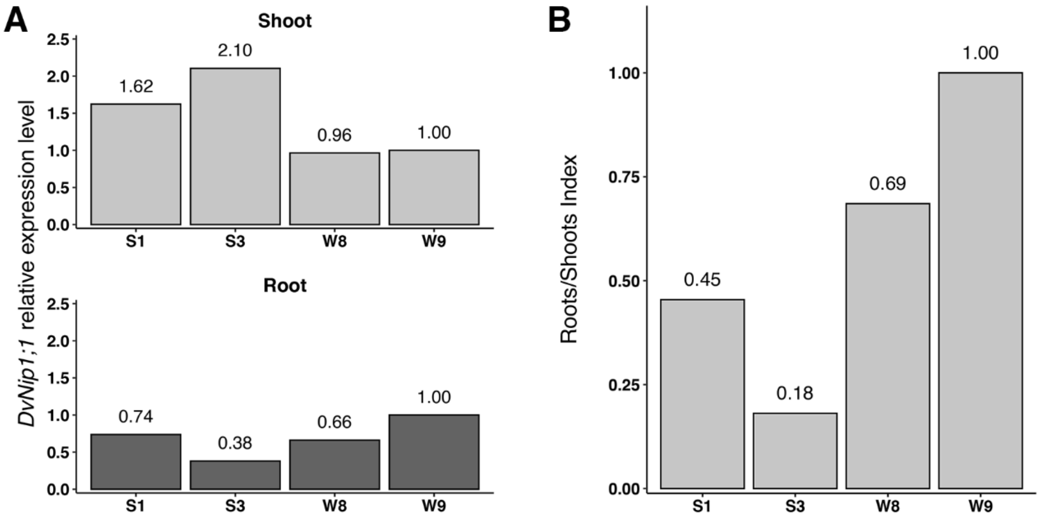

Figure S1. Relative expression level of DvNip1;1 in shoot and root calculated with the ΔΔCt technique (A) and ratio between the relative expression level of the gene in root and the relative expression level of the gene in shoot (B).

The W9 clone was one of the clones that show the highest Roots/Shoots Index value in the previous screening with the result that it is one of the less tolerant clone to As^{3+} stress. For this reason, here we used the expression level in root and shoot of W9 clone like reference for the calculation of the relative expression level. The resulting analysis enforce the importance for the clones to have a low Roots/Shoots Index to tolerate better the presence in the soil of HM and Metalloid like As^{3+} and Cd^{2+}.
